# Neprilysin inhibition promotes corneal wound healing

**DOI:** 10.1038/s41598-018-32773-9

**Published:** 2018-09-26

**Authors:** Rachel M. Genova, Kacie J. Meyer, Michael G. Anderson, Matthew M. Harper, Andrew A. Pieper

**Affiliations:** 1 Department of Molecular Physiology and Biophysics, University of Iowa Carver College of Medicine, Iowa City, IA USA; 2 Iowa City Department of Veterans Affairs Center for the Prevention and Treatment of Visual Loss, Iowa City, IA USA; 3 Department of Ophthalmology and Visual Sciences, University of Iowa Carver College of Medicine, Iowa City, IA USA; 4 Stephen A. Wynn Institute for Vision Research, Department of Ophthalmology and Visual Sciences, University of Iowa Carver College of Medicine, Iowa City, IA USA; 5 Department of Psychiatry, University of Iowa Carver College of Medicine, Iowa City, IA USA; 6 Harrington Discovery Institute, University Hospital Case Medical Center, Department of Psychiatry, Case Western Reserve University, Geriatric Research Education and Clinical Centers, Louis Stokes Cleveland VAMC, Cleveland, OH USA

## Abstract

Neprilysin (NEP), an ectoenzyme that modulates inflammation by degrading neuropeptides, was recently identified in the human corneal epithelium. The cornea expresses many NEP substrates, but the function of NEP in homeostatic maintenance and wound healing of the cornea is unknown. We therefore investigated the role of this enzyme under naive and injured conditions using NEP-deficient (NEP^−/−^) and wild type (WT) control mice. *In vivo* ocular surface imaging and histological analysis of corneal tissue showed no differences in limbal vasculature or corneal anatomy between naive NEP^−/−^ and WT mice. Histological examination revealed increased corneal innervation in NEP^−/−^ mice. In an alkali burn model of corneal injury, corneal wound healing was significantly accelerated in NEP^−/−^ mice compared to WT controls 3 days after injury. Daily intraperitoneal administration of the NEP inhibitor thiorphan also accelerated corneal wound healing after alkali injury in WT mice. Collectively, our data identify a previously unknown role of NEP in the cornea, in which pharmacologic inhibition of its activity may provide a novel therapeutic option for patients with corneal injury.

## Introduction

The cornea is a transparent dome-shaped structure that provides the majority of our optical refractive power and serves as a physical barrier against ocular pathogens^1^. As the outermost tissue of the eye, the cornea is vulnerable to injury, which is an underreported and globally significant cause of vision loss^2–4^. Chemical exposure causes severe ocular surface injury, and both acid and alkali corneal burns are ophthalmologic emergencies due to the rapidity of epithelial damage^5–7^.

During the first week after chemical injury, corneal reepithelialization occurs through epithelial cell migration, proliferation, and differentiation^8^. These processes are promoted by release of neuropeptides from a dense network of corneal nerves that arise from the trigeminal ganglia, in conjunction with growth factors and cytokines that are released locally by resident cells^9–11^. The resulting inflammatory reaction supports corneal nerve and epithelial regrowth after injury^12–14^. However, dysregulation of this process can lead to vision-damaging corneal ulceration, neovascularization, and opacification^15,16^.

If endogenous repair mechanisms fail to restore corneal transparency, reconstruction of the ocular surface after chemical injury is often attempted through surgical means. Though recent advances in autologous corneal stem cell transplantation can potentially restore a stable corneal epithelium without risk of rejection^17–20^, surgical outcomes are sensitive to tear film stability, intraocular pressure, and inflammation^21,22^. As a consequence, early medical interventions play an essential role in management of corneal injury and can ultimately be major determinants of surgical outcomes^15^. Indeed, for severe corneal injuries, visual prognosis frequently remains guarded, despite currently available medical and surgical techniques^6,23^. Development of alternative or complementary medical therapies is therefore critical for restoring corneal function and visual acuity after injury.

Membrane metallo-endopeptidase (commonly referred to as neprilysin and hereby abbreviated as NEP) is a widely distributed ectoenzyme that modulates inflammation by catalyzing degradation of vasoactive and neuroinflammatory peptides, including substance P, bradykinin, enkephalin, and atrial natriuretic factor^24,25^. This enzyme has gained considerable clinical attention as a drug target in cardiovascular disease, and safely-tolerated and efficacious inhibitors are already available for human use^26^. Five years prior to this study, NEP was identified in the human cornea^27^, a tissue that expresses many of its neuropeptide substrates. However, the function of NEP in the cornea is unknown, despite being an amenable therapeutic target.

In the present study, we investigate the role of NEP in the cornea under naive and alkali-injured conditions in order to determine whether it might participate in corneal homeostasis or recovery from injury. Using an NEP-deficient mouse, we demonstrate a previously unknown role of NEP in the cornea, where its deletion accelerates wound healing without affecting homeostatic maintenance. We further extend these findings by showing that the NEP inhibitor thiorphan also accelerates corneal wound healing in WT mice. Overall, we provide new insight into corneal wound healing mechanisms and identify NEP as a potential target to accelerate recovery and improve outcomes after corneal injury.

## Results

### NEP is functionally expressed in the mouse cornea

**Figure 1:**
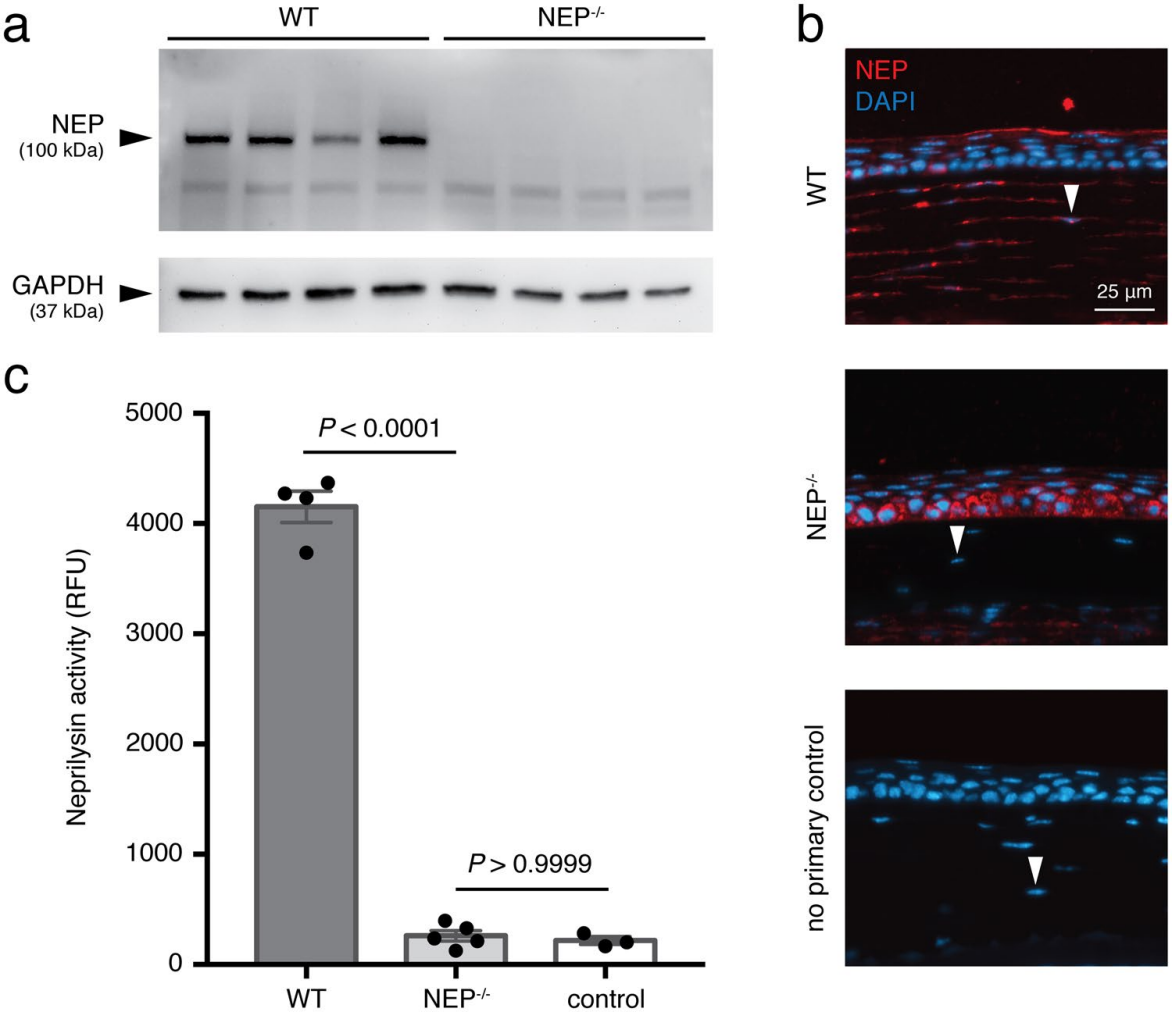
Functional expression of NEP in the mouse cornea. (**a**) Western blot showing expression of NEP in whole cornea lysates from WT and NEP^−/−^ mice with glyceraldehyde 3-phosphate dehydrogenase (GAPDH) run as a loading control on the same gel. Membrane was cut at 50 kDa. (**b**) Representative immunofluorescence images showing immunostaining for NEP (red) and DAPI (blue) in WT corneal sections (n = 3) compared to *NEP*^−/−^ (n = 3) and no primary control (n = 2) sections. NEP localizes to superficial epithelial cells and stromal keratocytes (arrowheads) in WT sections. Original magnification, 40×; scale bar, 25 μm. (**c**) NEP activity in whole cornea lysates, determined with a FRET-based assay (mean ± SEM; ****P < 0.0001; two-tailed *t*-test).

Western blot analysis of whole cornea lysates with anti-NEP antibody revealed the expected 100 kDa band in WT but not NEP^−/−^ tissue (Fig. 1a; see Supplementary Fig. 1 for full-length blot). With the same primary antibody, we then examined the cellular distribution of NEP with immunohistochemistry (Fig. 1b). In WT corneas, NEP was localized prominently in the apical epithelial layer and stromal keratocytes, with occasional signal in the cytoplasm of basal epithelial cells. Unexpectedly, NEP^−/−^ corneal sections also showed NEP staining, but in a different pattern with signal localizing to the basal epithelium in a perinuclear distribution. Similar patterns of staining in NEP^−/−^ tissue were observed using other monoclonal and polyclonal antibodies against NEP (data not shown). No signal was detected in the no primary antibody controls. The reason for this immunopositive signal in NEP^−/−^ mice is unknown. Conceivably, expression in the NEP^−/−^ mouse could reflect a truncated protein resulting from exonic cassette insertion when the mouse was generated. In order to establish whether this signal in NEP^−/−^ mice represented functional NEP protein, we assayed for NEP activity in whole cornea lysates. Activity in WT samples was readily detected at levels significantly above that in NEP^−/−^ samples (Fig. 1c, *P* < 0.0001). Signal from NEP^−/−^ samples, however, did not exceed that in negative control wells (Fig. 1c, *P* > 0.9999). Thus, the NEP signal seen on histology in NEP^−/−^ tissue represents either non-specific staining or an inactive, truncated form of NEP.

### NEP deficiency is associated with normal ocular surface and corneal thickness

**Figure 2:**
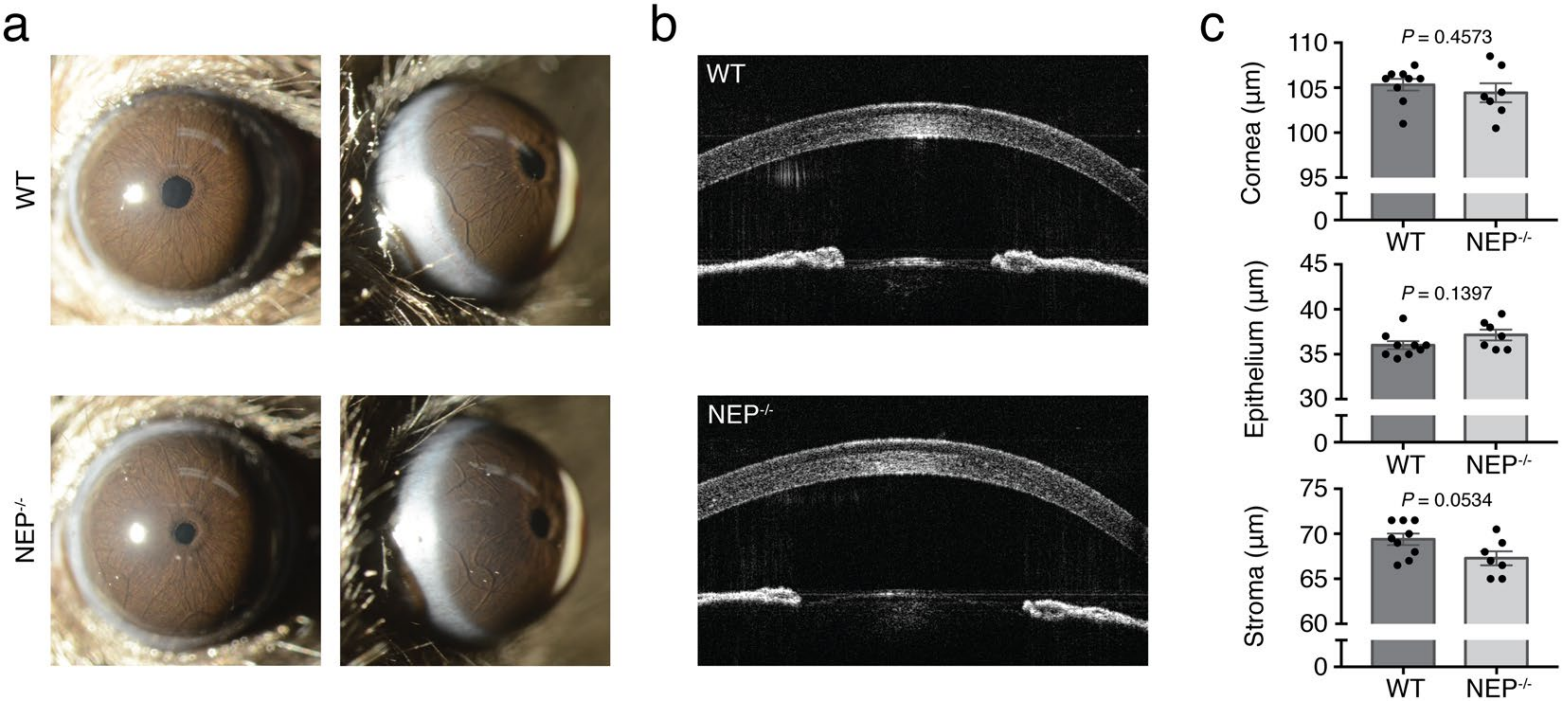
Gross corneal phenotype of NEP^−/−^ mice. (**a**) Broad beam slit lamp images of WT and NEP^−/−^ eyes showing corneal surfaces and limbal regions (n = 3 mice/group). (**b**) Representative images of WT and NEP^−/−^ corneas obtained with anterior segment optical coherence tomography. (**c**) Quantification of central corneal thickness and thickness of epithelial and stromal layers (n = 7–9 mice/group; mean ± SEM; two-tailed *t*-test).

To assess the effect of constitutive NEP disruption on corneal morphology, we used two *in vivo* imaging techniques: slit lamp biomicroscopy, which uses an adjustable beam of incandescent light to image the eye from various angles; and anterior segment optical coherence tomography (OCT), which uses long wavelength light to non-invasively capture micrometer-resolution images of the eye. Non-invasive *in vivo* imaging allows examination of corneal morphology in the absence of artifacts that are typically introduced during tissue processing for histology. No epithelial defects, cataracts, corneal opacities, or corneal neovascularization were observed on slit lamp examination of awake, unmanipulated WT or NEP^−/−^ mice (Fig. 2a). Central corneal thickness (CCT), epithelial thickness, and stromal thickness were then quantified using OCT (Fig. 2b). No significant differences were detected in CCT or epithelial thickness between groups, though there was a trend for increased stromal thickness in NEP^−/−^ corneas compared to WT controls (Fig. 2c, *P = *0.0534).

### NEP^−/−^ mice have increased corneal innervation and normal pericorneal vasculature

**Figure 3:**
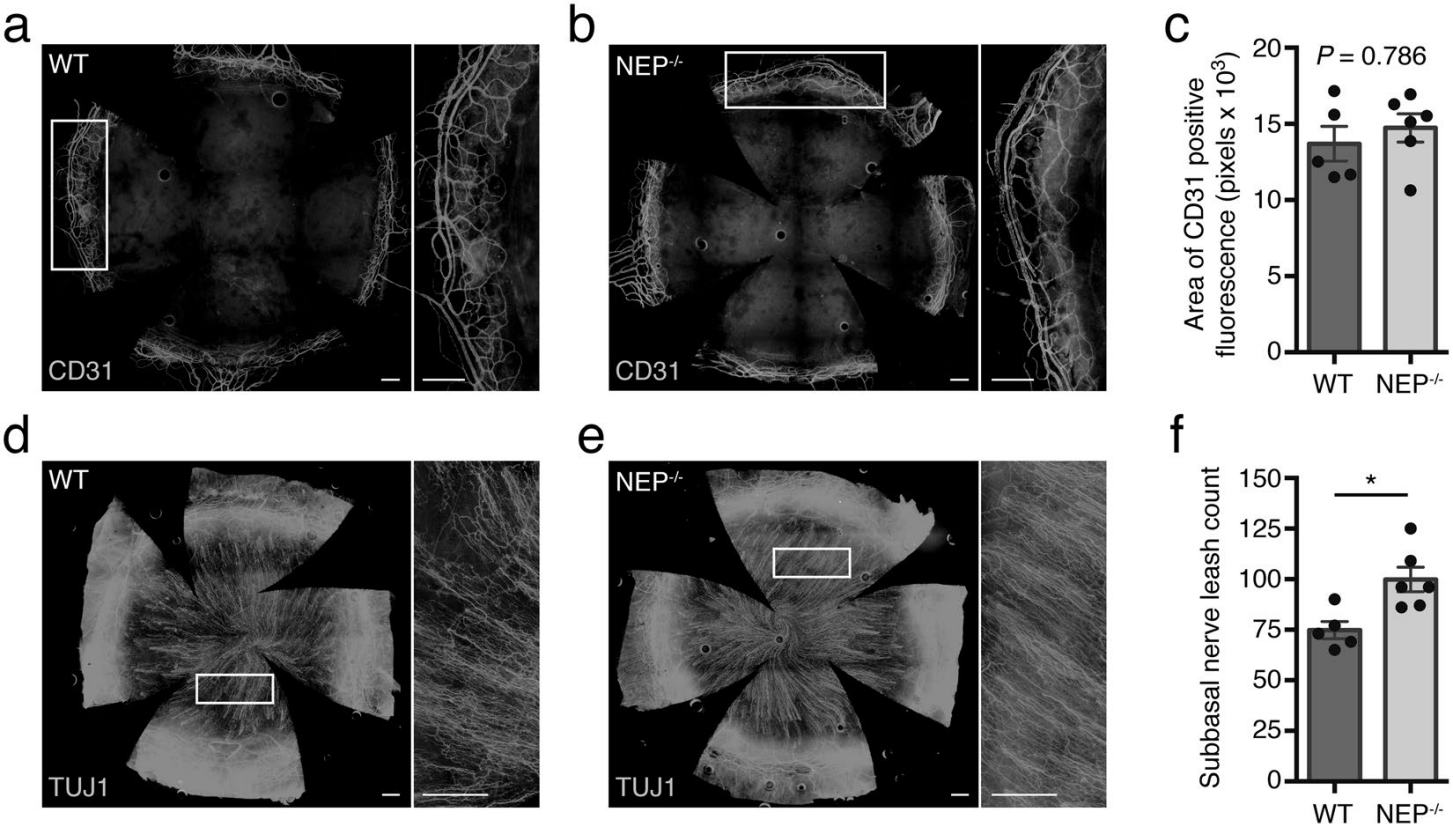
Limbal vasculature and corneal innervation in WT and NEP^−/−^ mice. Representative corneal flat mounts from a WT (**a**) and NEP^−/−^ (**b**) mouse immunostained with the vascular marker CD31. Higher magnification images of boxed areas in (**a**) and (**b**) are shown to the right of each image, respectively. (**c**) Area of positive CD31 immunofluorescence (n = 5–6 mice/group, mean ± SEM; two-tailed *t*-test). Representative corneal flat mounts from a WT (**d**) and NEP^−/−^ (**e**) mouse immunostained with the pan-neuronal marker βIII tubulin (TUJ1). Higher magnification images of boxed areas in (**d**) and (**e**) are shown to the right of each image, respectively. (**f**) Subbasal nerve leashes (n = 5–6 mice/group, mean ± SEM; *P = 0.0103; two-tailed *t*-test). Original magnification, 10×; scale bar, 250 μm for all images.

To further assess the pericorneal vasculature, we immunolabeled whole mount corneas for the endothelial marker CD31 to visualize blood vessels. As expected, the pericorneal plexus was appropriately confined to the limbal area and did not encroach onto the peripheral cornea in either WT or NEP^−/−^ mice (Fig. 3a,b). Quantification of CD31 staining confirmed that there was no significant difference in the total area of vasculature between groups (Fig. 3c, *P = *0.786). In the same corneas, we also immunolabeled for βIII tubulin (TUJ1) to visualize corneal innervation at the level of the subbasal nerve plexus (Fig. 3d,e), which is composed of radially-oriented unmyelinated fibers that run beneath the basal epithelium and terminate between superficial epithelial cells^28^. Notably, NEP^−/−^ corneas had 33.47 ± 8.11% more subbasal nerve leashes than WT corneas (Fig. 3f, *P* = 0.0103).

### NEP deficiency promotes corneal epithelial wound healing *in vivo*

**Figure 4:**
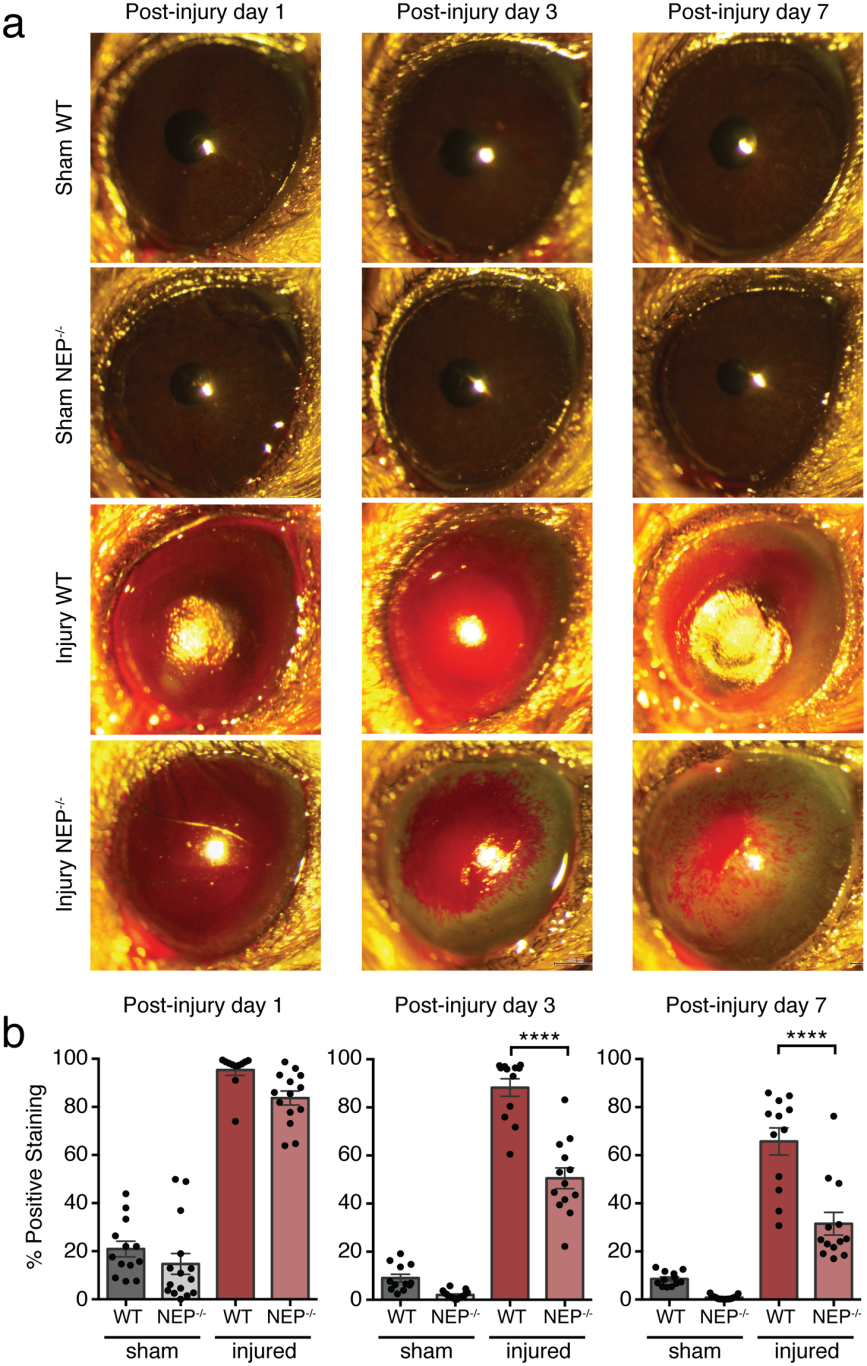
Corneal wound healing after alkali burn in WT and NEP^−/−^ mice. (**a**) Representative broad beam slit lamp images obtained at 1, 3, and 7 days post-injury with rose bengal instillation to visualize corneal defects. (**b**) Quantification of positive rose bengal staining reported as a percentage of total corneal area at 1, 3, and 7 days post-injury (n = 12–18 mice/group at each timepoint; mean ± SEM; ****P < 0.0001; one-way ANOVA with Bonferroni correction).

To evaluate the effect of NEP deficiency on corneal wound healing, we chemically injured the corneas of NEP^−/−^ and WT mice with controlled application of sodium hydroxide to the ocular surface. This method of injury reproducibly resulted in a grade III burn according to Roper-Hall criteria for corneal involvement (Supplementary Fig. 2). The acute phase of wound healing was then tracked with slit lamp imaging at 1, 3, and 7 d after injury, with rose bengal staining serving as a measure of the corneal defect at each timepoint (Fig. 4a). The corneas of sham-injured mice retained minor rose bengal stain after sham injury, typically in the central and/or nasal cornea. However, the pattern of dye retention was superficial compared to that observed in alkali-injured animals, and sham-injured corneas retained clarity and regularity of the ocular surface over the 7 days post-injury (Fig. 4a). There was also no significant difference in the area of positive staining between WT and NEP^−/−^ sham groups at any timepoint (Fig. 4b). In the alkali-injured groups, corneas appeared diffusely opaque and edematous at 1 day post-injury. At this acute time point, the area of corneal defect in alkali-injured NEP^−/−^ mice was not significantly different from that in alkali-injured WT mice (Fig. 4b, left panel). However, at 3 and 7 days post-injury, the corneal defect area in NEP^−/−^ mice was 42.72 ± 4.91% and 52.08 ± 7.19% smaller, respectively, than that in WT mice (Fig. 4b, middle and right panels, *P* < 0.0001 at both time points).

### The NEP inhibitor thiorphan promotes corneal epithelial wound healing *in vivo*

Based on the accelerated wound healing phenotype that we observed in NEP^−/−^ mice, we then evaluated efficacy of the NEP inhibitor thiorphan on corneal wound healing in WT mice. First, to determine whether intraperitoneal (*i.p*.) thiorphan could inhibit NEP activity in the cornea, we assayed for NEP activity in whole cornea lysates from uninjured mice. Because there are mixed reports about whether parenteral thiorphan can cross the blood-brain barrier^29^, we also isolated trigeminal ganglia from the same animals to determine whether thiorphan could inhibit NEP activity in the cell bodies of neurons innervating the cornea. A single *i.p*. dose of 15 mg/kg thiorphan in an uninjured WT mouse decreased NEP activity in the cornea by 58.26 ± 8.96% at 1 h after administration (n = 3 mice, *P* = 0.0226) and by 34.14 ± 10.43% at 6 hours after administration (n = 3 mice, *P* = 0.1407), compared to vehicle controls (Supplementary Fig. 3). The same dose decreased NEP activity in the trigeminal ganglia by 28.39 ± 1.65% (n = 3 mice, *P* = 0.002) and 8.18 ± 4.24% (n = 3 mice, *P* = 0.3243) at 1 and 6 hours after administration, respectively, compared to vehicle controls (Supplementary Fig. 3).

**Figure 5:**
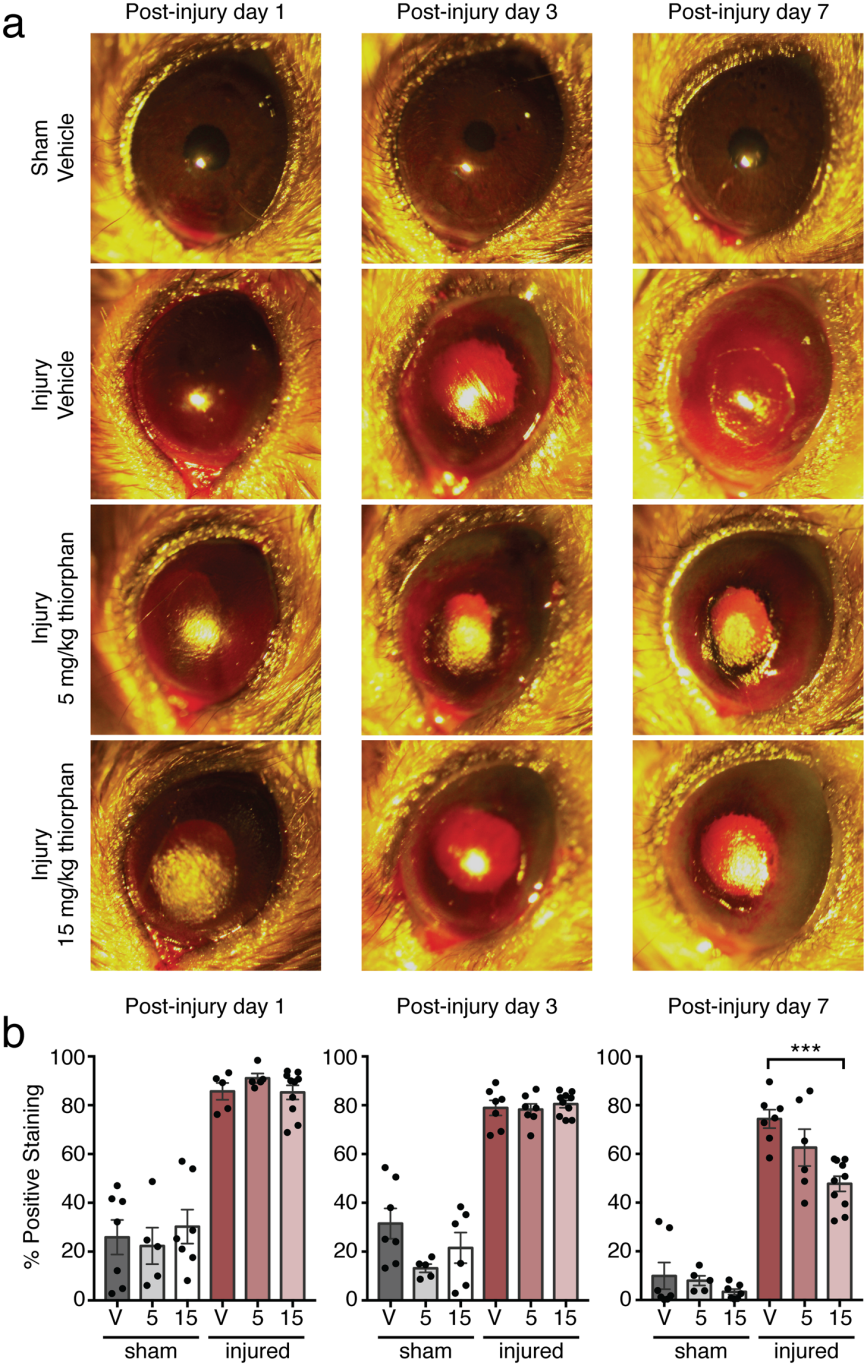
Corneal wound healing after alkali burn with thiorphan administration in WT mice. (**a**) Representative broad beam slit lamp images obtained at 1, 3, and 7 days post-injury with rose bengal instillation to visualize corneal defects. (**b**) Quantification of positive rose bengal staining reported as a percentage of total corneal area at 1, 3, and 7 days post-injury (n = 5–10 mice/group at each timepoint; mean ± SEM; ***P = 0.0004; one-way ANOVA with Bonferroni correction). V, vehicle; 5, 5 mg/kg thiorphan; 15, 15 mg/kg thiorphan.

In our chemical corneal injury model, we then administered *i.p*. thiorphan at 5 or 15 mg/kg daily to WT mice. Representative slit lamp images are shown in Fig. 5a. There were no significant differences in the area of positive rose bengal staining between vehicle, low dose, or high dose thiorphan-treated groups at the early time points of 1 or 3 days post-injury (Fig. 5b, left and middle panels). At 7 days post-injury, however, the high dose thiorphan group had corneal defects 35.80 ± 4.20% smaller than those in vehicle-treated mice (Fig. 5b, right panel, *P* = 0.0004). There was no significant difference in corneal defect area between the low and high dose thiorphan groups (*P = *0.2305), or between the low dose thiorphan and vehicle groups (*P* > 0.999). By 7 days post-injury, two mice in the vehicle-treated group (n = 8) and three in the low dose thiorphan-treated group (n = 8) required euthanization due to corneal perforation. None of the high dose thiorphan-treated mice (n = 10) developed corneal perforation (Supplementary Fig. 4).

Though the cornea may initially undergo reepithelialization after corneal burn, severe injury may result in vision-compromising sequelae, including stromal opacity and abnormal vascular growth^15^. To determine if NEP inhibition acutely affects inflammation, corneal neovascularization, or fibrosis after alkali injury, we immunoblotted for markers of leukocytic infiltration (CD45), vascular endothelium (CD31), and myofibroblastic cells (alpha-smooth muscle actin) in corneas from alkali- and sham-injured mice treated with 15 mg/kg thiorphan or vehicle (Supplementary Fig. 5). Expression of these markers at 1 week post-injury in thiorphan-treated mice (n = 7) did not significantly differ from vehicle-treated mice (n = 7; CD45, P > 0.999; CD31, P* = *0.080; alpha-smooth muscle actin, P = 0.769). The immunoblots were repeated with similar results.

Based on the effects of thiorphan on corneal reepitheliazation *in vivo*, we utilized an *in vitro* wound healing assay to begin dissecting the mechanism of accelerated corneal resurfacing with NEP inhibition. Expression of NEP in TKE2 cells, a line of mouse corneal epithelial progenitors isolated from a CD-1 female, was first confirmed by immunoblot (Supplementary Fig. 6). Cultures were then grown to confluence, wounded, treated with thiorphan or vehicle, and imaged at intervals for 18 h (Supplementary Fig. 7a). In this cell migration-based assay, thiorphan did not enhance the rate of wound closure compared to vehicle controls (Supplementary Fig. 7b,c; n = 5 wells per condition; P = 0.6436). Data are representative of two independent assays.

## Discussion

In the present study, we report functional expression of NEP in the mouse cornea and a critical role for NEP in corneal wound healing. Previously, a single study described NEP expression in human corneal epithelium^27^, but its physiological role was unknown. Our examination of the adult mouse cornea with *in vivo* imaging and histology has now revealed that gross corneal morphology, epithelial and stromal thickness, and perilimbal vasculature are all unaltered by NEP deficiency. However, we did observe increased corneal innervation in NEP^−/−^ mice, based on subbasal nerve leash number. This is in congruence with a recent study on the effect of NEP on diabetic neuropathy in which Yorek *et al*.^30^ reported a trend of increased corneal nerve density in nondiabetic NEP^−/−^ mice, compared to WT controls. Collectively, our findings demonstrate that NEP is not essential for homeostatic maintenance of corneal epithelium, but may be involved in regulating development and/or maintenance of corneal nerves.

Indeed, multiple studies have suggested that NEP regulates peripheral nerve development. Expressed on the plasma membranes of both myelin-forming and non-myelin-forming Schwann cells in prenatal and early postnatal development^31^, NEP levels decline in myelin-forming Schwann cells as myelination concludes in adulthood, paralleling the developmental profile of other axonally-controlled antigens^32^. Though the substrate(s) for NEP on Schwann cell membranes are unknown, interleukin-1 (IL-1) has been suggested as a potential candidate. Macrophages closely associate with peripheral nerves in neonatal life^33^ and function as a source of IL-1 to stimulate nerve growth factor (NGF) production by Schwann cells^34^. NEP may therefore compete with IL-1 receptors on Schwann cell membranes to modulate NGF production. In this case, loss of NEP activity would increase NGF production and perhaps account for the increased corneal innervation observed in NEP^−/−^ mice. As a cell surface peptidase readily detected in uninjured corneal epithelium, NEP is well-positioned to modulate peptidergic crosstalk between corneal epithelial cells and corneal nerves as well, though additional work is needed to discern the origin of this difference in innervation.

Because the interaction between corneal nerves and epithelial cells is critical for wound healing^35–39^, we focused here on investigating the effect of acutely modulating NEP after corneal injury. For this, we applied a model of alkali burn, a severe form of chemical injury with an unmet need for improved pharmacologic treatments^5,7^. Alkaline agents are commonly found in various household cleaning products, including bleach, oven cleaners, and drain cleaners. Consequently, corneal alkali injuries occur more often and also cause more damage than their acid counterparts, with risk to young children substantially higher than previously estimated^4^.

In contrast to mechanical abrasions and other epithelial injuries that can resurface within a week, alkali injury is seldom restricted to the epithelium. Because their cationic form is lipophilic, alkaline chemicals rapidly penetrate the ocular surface and can damage all corneal layers, as well as the lens, ciliary body, and trabecular meshwork^15,40^. Recovery, if possible, occurs over weeks to months. This protracted course is also encountered in rodents^41^. However, after chemical corneal injury, rapid reepithelialization is necessary to restore the protective barrier of tight junctions and limit recruitment of inflammatory cells, thereby reducing the risk of infection, scarring, persistent corneal defects, and other sight-threatening sequelae. Clinically, the degree of corneal epithelial recovery correlates with patient prognosis after chemical injury, with earlier resurfacing predictive of improved outcomes^15,42^. For this reason, we evaluated recovery in our mouse model of alkali corneal burn during the first week post-injury to coincide with the acute phase of corneal wound healing.

During this post-injury period, we observed that corneal epithelial wound healing in NEP^−/−^ mice was significantly accelerated relative to WT mice. When comparing corneal resurfacing in WT animals to those with constitutive loss of NEP activity, a potential confounding factor is the increased density of corneal innervation observed in NEP^−/−^ mice. Because corneal nerves produce epitheliotrophic factors that promote physiological renewal as well as wound healing^43–45^, accelerated corneal wound healing in NEP^−/−^ mice may be related to increased innervation at baseline. However, pharmacologic NEP inhibition with thiorphan also promoted epithelial recovery in WT mice within one week of corneal injury, an interval too brief for extensive reinnervation. This suggests that a difference in innervation, whether pre- or post-injury, is likely not the primary mechanism by which genetic or pharmacologic disruption of NEP activity supports corneal wound healing at the acute time points investigated here. However, at one week post-injury, there was a trend for decreased expression of the vascular marker CD31 in corneas from thiorphan-treated mice, indicating delayed or reduced development of neovascularization after alkali burn. Given that nerves and neovessels reciprocally inhibit each other in the cornea^46^, thiorphan could indirectly support corneal nerve regrowth at later stages of repair through reduction of neovascularization. Longer-term studies are necessary to evaluate the extent and mechanism of this effect.

Thiorphan is a highly potent and specific NEP inhibitor, with an IC50 reported as low as 1.8 nM^47,48^. In addition, it is the active metabolite of racecadotril, an agent used clinically for hypersecretory diarrhea in Europe, Asia, and South America^29,49^. Though racecadotril is not available for use in the United States, the NEP inhibitor sacubitril is currently approved for management of chronic heart failure^26^. This positions our basic science findings for rapid clinical translation. Furthermore, efficacy of thiorphan administration in this model of corneal alkali injury suggests that NEP inhibition may have therapeutic utility in other types of corneal injury, such as mechanical abrasions or damage associated with dry eye disease. These, along with the effect of NEP inhibition on long-term outcomes of alkali injury, are avenues of future investigation.

Corneal epithelial repair during the acute phase of wound healing involves coordination of cellular, molecular, and functional changes in viable tissue at the wound margin. Local release of cytokines, growth factors, and neuropeptides facilitates reciprocal communication between the reparative epithelium and neighboring stromal keratocytes, corneal nerves, and immune cells to coordinate clearance of cellular debris from the wound bed, synthesis of adhesion proteins and extracellular matrix, and corneal epithelial cell migration and proliferation^9–11,50^. The particular role of NEP in this complicated cascade is unknown. Inhibition of NEP could reduce the enzymatic degradation of numerous growth factors and neuropeptides^24,25,51–53^, thereby prolonging their actions and accounting for accelerated corneal epithelial wound healing in NEP^−/−^ and thiorphan-treated WT mice.

As an initial step towards unraveling the mechanism of NEP inhibition in corneal wound healing, we employed an *in vitro* model of epithelial injury. Scratch assays with the TKE2 cell line suggest that modulation of NEP activity does not alter epithelial cell migration after wounding. Alternatively, NEP inhibition may require the presence of other cell types, such as stromal keratocytes, immune cells, or sensory neurons, in order to alter epithelial behavior. An example of this interdependence is seen with substance P (SP) *in vivo*. Predominantly produced and released by sensory nerves in the cornea^54,55^, SP sensitizes the corneal epithelium to circulating trophic factors produced elsewhere in the body^56–58^. Loss of innervation, and thus the primary source of SP, renders the corneal epithelium unresponsive to physiologic concentrations of these agents^35^. Conceivably, the results of our scratch assays may therefore be an artifact of the isolated system in which thiorphan was tested. Still, this alternative interpretation presupposes that the therapeutic action of thiorphan occurs through direct interaction with corneal tissue. It may be that thiorphan acts peripherally on immune cells or the endothelium of pericorneal vasculature, thereby modulating the wound environment to indirectly support reepithelialization.

Although further mechanistic investigation is warranted, our findings provide robust and novel preclinical support for a new pharmacologic approach to managing corneal injury, as well as potentially injury to other epithelial tissues, with promise for clinical translation using existing NEP inhibitors. Combination of NEP inhibitors with standard medical treatments for alkali burn, such as topical ascorbate, citrate, antibiotics, and steroids^7^, may assist in optimizing the corneal surface for reepithelialization, thereby mitigating the risk of sight-threatening complications in the later stages of wound healing.

## Methods

### Animals

Eight to twelve week old male mice were used for all experiments. Breeding pairs of mice with a targeted disruption of the membrane metallo-endopeptidase gene (B6.129S4-*Mme*^*tm1Cge*^; abbreviated throughout as NEP^−/−^) were provided by Drs. Lu and Gerard^59^ and bred to establish colonies at the Iowa City Department of Veterans Affairs and University of Iowa (Iowa City, IA) in certified animal care facilities. Offspring were backcrossed to C57BL/6J mice for more than nine generations. Control C57BL/6J mice (The Jackson Laboratory, Bar Harbor, ME) were also housed at these locations. Standard diet (Harlan Teklad, #7001, Madison, WI, USA) and water were provided *ad libitum* at both facilities. Mice were handled in accordance with the ARVO Statement for the Use of Animals in Ophthalmic and Vision Research, and protocols were approved by the Institutional Animal Care and Use Committees at the Iowa City Department of Veterans Affairs and University of Iowa.

### Western blot analysis

Freshly enucleated eyes were trimmed at the sclerocorneal limbus. Corneas from each mouse were pooled and homogenized (VWR 200 Homogenizer, Radnor, PA, USA) in 100 μL cold RIPA buffer containing protease/phosphatase inhibitors (Halt Cocktail; Thermo Scientific Pierce Biotechnology, Rockford, IL, USA). Total protein was determined by BCA assay (Thermo Scientific). Corneal lysates containing equal amounts of protein (15 μg) were separated on a 10% Mini-PROTEAN TGX precast polyacrylamide gel (BioRad, Hercules, CA, USA) and transferred to a nitrocellulose membrane with the Trans-Blot Turbo Transfer system (BioRad, Hercules, CA, USA). Membranes were cut at 50 kDa and blocked in 5% skim milk in TBST buffer at room temperature (RT) for 1.5 h. The upper half of the membrane (>50 kDa) was immunoblotted in goat anti-CD10 (NEP; PA5-47075; Invitrogen) at 1:100, and the lower half (<50 kDa) was immunoblotted in mouse anti-GAPDH (loading control; MAB374; EMD Millipore, Burlington, MA, USA) at 1:1000, both overnight at 4 °C. After washing in TBST, membranes were incubated with HRP-conjugated secondary antibodies (Abcam, Cambridge, UK) at 1:5000 for 1 h at RT and developed with SuperSignal West Femto Maximum Sensitivity Substrate (Thermo Scientific). Chemiluminescence was detected using a BioSpectrum 810 imaging system with CCD camera (Ultra-Violet Products, Upland, CA, USA).

### NEP enzyme activity

Corneas from each mouse were pooled, frozen in liquid nitrogen, and transferred to −80 **°**C storage. Frozen samples were homogenized in 100 μL of 0.5% NP-40 lysis buffer in two 15 s bursts with 30 s on ice between bursts. Aprotinin (5 μg/mL; Thermo Scientific) and phenylmethylsulfonyl fluoride (200 μM; Thermo Scientific) were added to the buffer to prevent protein degradation. Total protein in each sample was determined by BCA assay according to manufacturer’s instructions (Thermo Scientific).

NEP activity was determined using the SensoLyte 520 Neprilysin Activity Assay Kit (AnaSpec, Fremont, CA, USA). Samples were diluted in 1x assay buffer to 15 μg protein/50 μL/well and loaded in triplicate in 96-well plates. Positive, negative, and substrate controls were run according to manufacturer’s protocol. Plates were incubated and read at 24 **°**C. Endpoint fluorescence was recorded on a SpectraMax M2 microplate reader (Molecular Devices, Sunnyvale, CA, USA) with excitation at 490 nm and emission at 520 nm, per manufacturer’s protocol. The assay was performed twice on different sets of tissue samples. Background-subtracted data are presented from a single assay.

### Immunofluorescence and microscopy

For immunofluorescent localization of NEP, freshly enucleated eyes were fixed whole in 4% paraformaldehyde for 30 min at RT prior to routine dehydration and paraffin-embedding. Eyes were sectioned sagittally at 5 μm intervals, mounted on Fisherbrand Superfrost Plus slides, deparaffinized, and rehydrated. For antigen retrieval, sections were placed in a IHC-Tek Epitope Retrieval Steamer (IHC World, Ellicott City, MD, USA) for 45 min in Citra Plus Antigen Retrieval Solution (BioGenex, Fremont, CA, USA). Sections were then incubated in blocking buffer (5% BSA in 0.1% Triton X-PBS) for 2 h at RT. Goat anti-CD10 (NEP; PA5-47075; Invitrogen) was applied to slides with CoverWell Incubation Chamber Gaskets (Thermo Scientific) at 1:20 in blocking buffer overnight at 4 **°**C. Negative controls were incubated in buffer only. After washing in 0.1% Tween, sections were incubated with an Alexa Fluor 647-conjugated secondary antibody (Abcam) at 1:500 for 2 h at RT. Finally, slides were washed and coverslipped with ProLong Diamond Antifade Mountant with DAPI (Thermo Scientific). Slides were imaged on a Zeiss Axio Imager 2 at 40X with identical exposure settings for all sections.

For corneal whole mount CD31 and TUJ1 immunostaining, enucleated eyes were fixed in Zamboni’s (Newcomer Supply, Middleton, WI, USA) for 30 min at RT. Corneas were dissected at the sclerocorneal limbus and incubated in blocking buffer (2% BSA, 2% normal donkey serum, 0.2% Triton-X) for 2 h at RT. Corneas were incubated in antibodies against endothelial adhesion marker CD31 and pan-neuronal marker βIII tubulin/TUJ1 (rabbit anti-CD31 at 1:200, Abcam; mouse anti-TUJ1 at 1:1000, Biolegend, San Diego, CA, USA) overnight at 4 **°**C. After washing in 0.1% Tween, corneas were incubated with Alexa Fluor 488- and 647-conjugated anti-rabbit and anti-mouse secondary antibodies (Abcam), respectively, at 1:500 for 2 h at RT. Corneas were washed in 0.1% Tween and mounted on glass slides as above.

Tiled images were taken on an Olympus BX-61 motorized microscope at 10X with identical exposure settings for all corneas, and stitched using Olympus cellSens software. The area of positive CD31 staining was measured in ImageJ (NIH) using thresholding and region of interest functions. Subbasal nerve leashes were counted manually in ImageJ.

### *In vivo* ocular surface imaging and ocular examination

The ocular surfaces of conscious mice were examined under a slit lamp microscope (SL-D7; Topcon, Tokyo, Japan). Images were taken with a digital camera (D800; Nikon, Tokyo, Japan) using identical settings.

A separate cohort of mice was anesthetized with *i.p*. ketamine/xylazine (100 mg ketamine + 10 mg xylazine/kg body weight) for bilateral measurement of central corneal thickness (CCT) with spectral domain optical coherence tomography (SD-OCT; Bioptigen, Durham, SC, USA). Scans of the anterior segment were obtained with a 12 mm telecentric bore centered over the pupil, with the following parameters: 2.0 mm radial volume scans, 1000 A scans/B scan, 100 B scans/volume, 1 frame/B scan, 1 volume. CCT, epithelial thickness, and stromal thickness were measured with vertical-locked B scan calipers in the Bioptigen InVivoVue Clinic software. Mice were omitted from analysis if variation in CCT between left and right eyes exceeded 5 μm.

### Alkali burn model of corneal injury

Prior to wounding, mice were anesthetized with *i.p*. ketamine/xylazine and received topical 0.5% proparacaine (Bausch + Lomb, Rochester, NY, USA) for corneal analgesia. A single 5 μL drop of 0.5 M NaOH was applied unilaterally to the ocular surface for 30 s, followed by immediate irrigation with 5 mL isotonic saline. A unilateral, saline instillation served as a sham injury. Ocular surfaces were coated with a 2.5% methylcellulose ophthalmic lubricant (Goniovisc, HUB Pharmaceuticals, Rancho Cucamonga, CA) to prevent drying and reduce infection risk after injury. Animals were monitored until ambulatory. Subcutaneous meloxicam (2 mg/kg; Newbrook, Newry, Northern Ireland) was provided up to 48 h after injury for pain control. All animals were euthanized by rapid decapitation under isoflurane anesthesia 7 d after injury.

### Analysis of wound closure

Corneal wounds were visualized with 0.1% rose bengal (Sigma-Aldrich, St. Louis, MO, USA) under a slit lamp microscope (Zeiss) equipped with a digital camera (Olympus DP21). Duplicate images were acquired of the ocular surface at 1, 3, and 7 d post-injury under ketamine-xylazine anesthesia. During each anesthetic event, eyes were protected by a sterile, water-soluble ophthalmic lubricant (Optixcare; CLC Medica, Waterdown, Ontario, Canada) to prevent drying of the ocular surface before and after imaging.

Quantitative analyses of corneal rose bengal staining were performed using a custom-written color deconvolution algorithm in Aperio Spectrum software (Leica Biosystems, Buffalo Grove, IL, USA). Regions of reflected light were omitted from the analyzed area. Total percent staining within a user-defined area (here, the cornea) was averaged for each pair of images. Data from all corneal injury studies are representative of at least two independent experiments.

### Thiorphan preparation and administration

Stock solutions of 30 mg/mL DL-thiorphan (N1195; Bachem, Bubendorf, Switzerland) were prepared in 100% ethanol and stored at −20 **°**C. Working solutions of 1.5 mg/mL were prepared daily by dilution in isotonic saline. Saline containing 5% ethanol served as a vehicle control. Intraperitoneal 5 mg/kg or 15 mg/kg thiorphan, or vehicle was administered to mice within 1 h after corneal injury. Administration was continued daily in each group until euthanasia on day 7 after injury.

### Statistics

Results are presented as mean ± SEM. Student’s unpaired *t* test was used to compare CCT, corneal epithelial thickness, stromal thickness, positive CD31 immunostaining, and subbasal nerve leash count between groups. A one-way ANOVA with *post hoc* Bonferroni correction for multiple comparisons was used to compare NEP activity and corneal rose bengal staining among groups. All statistical analyses were performed in Graphpad Prism 7 (San Diego, CA, USA). Throughout, *P* ≤ 0.05 was considered statistically significant.

## Electronic supplementary material

Supplementary Information and Data Figures

## Data Availability

The datasets generated during and/or analysed during the current study are available from the corresponding author on reasonable request.
