# Supplementary material for: Neprilysin inhibition promotes corneal wound healing
Source: Sci Rep. 2018 Sep 26;8:14385. doi: 10.1038/s41598-018-32773-9 (PMC6158251; doi:10.1038/s41598-018-32773-9)
Supplement: Supplementary file 1 — Supplementary Information and Data Figures [file 41598_2018_32773_MOESM1_ESM.pdf]

Neprilysin inhibition promotes corneal wound healing

Rachel M. Genova[1], Kacie J. Meyer[1,2,3,4], Michael G. Anderson[1,2,3,4], Matthew M. Harper*[2,3], Andrew A. Pieper*[1,2,3,4,5,6]

[1] Department of Molecular Physiology and Biophysics, University of Iowa Carver College of Medicine, Iowa City, IA, USA

[2] Iowa City Department of Veterans Affairs Center for the Prevention and Treatment of Visual Loss, Iowa City, IA, USA

[3] Department of Ophthalmology and Visual Sciences, University of Iowa Carver College of Medicine, Iowa City, IA, USA

[4] Institute for Vision Research, Department of Ophthalmology and Visual Sciences, University of Iowa Carver College of Medicine, Iowa City, IA, USA

[5] Department of Psychiatry, University of Iowa Carver College of Medicine, Iowa City, IA, USA

[6] Harrington Discovery Institute, University Hospital Case Medical Center; Department of Psychiatry, Case Western Reserve University; Geriatric Research Education and Clinical Centers, Louis Stokes Cleveland VAMC Cleveland, OH, USA

*Correspondence to:

Andrew A. Pieper, MD PhD, Harrington Discovery Institute, University Hospital Case Medical Center; Department of Psychiatry, Case Western Reserve University; Geriatric Research Education and Clinical Centers, Louis Stokes Cleveland VAMC, Cleveland, OH, USA
Email: AndrewAPieper@gmail.com

Or

Matthew M. Harper, PhD, Iowa City Department of Veterans Affairs Center for the Prevention and Treatment of Visual Loss, Iowa City, IA, USA; Department of Ophthalmology and Visual Sciences, University of Iowa Carver College of Medicine, Iowa City, IA, USA
Email: matthew.harper@va.gov

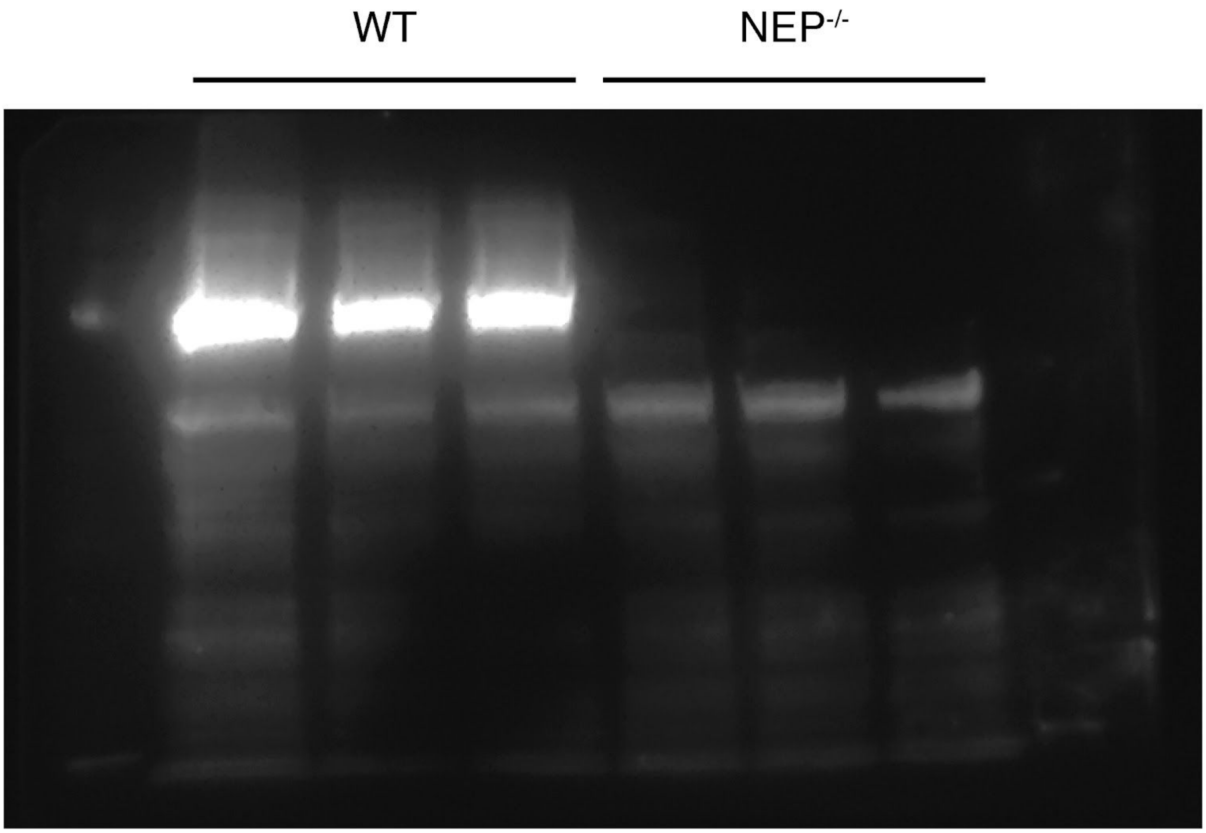

Supplementary Figure 1. Full length blot for NEP in whole cornea lysates. Western blot showing expression of NEP in whole cornea lysates from WT and NEP$^{-/-}$ mice (n = 3). NEP bands (100 kDa) in WT lanes are overexposed to allow visualization of additional bands.

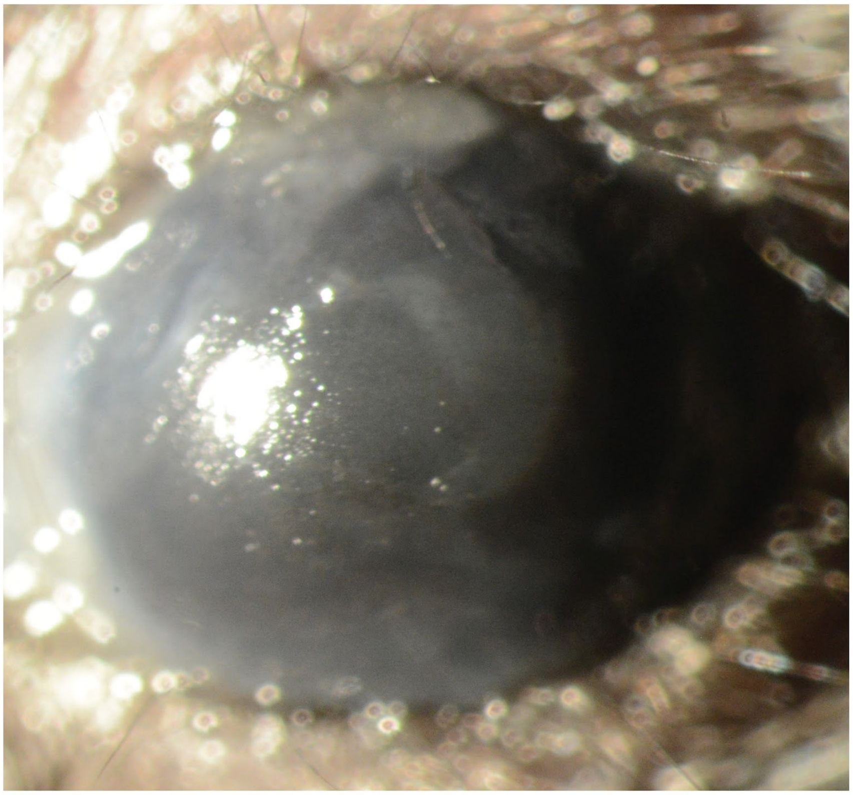

Supplementary Figure 2. Broad-beam slit lamp image of the alkali-injured cornea. Representative image of the ocular surface 1 day after alkali injury, without rose bengal instillation to illustrate the density and area of corneal haze. Details of the iris and pupil are obscured, but the pupil remains distinguishable from the iris. This injury represents a grade III burn according to Roper-Hall criteria for corneal involvement; limbal ischemia was not determined.

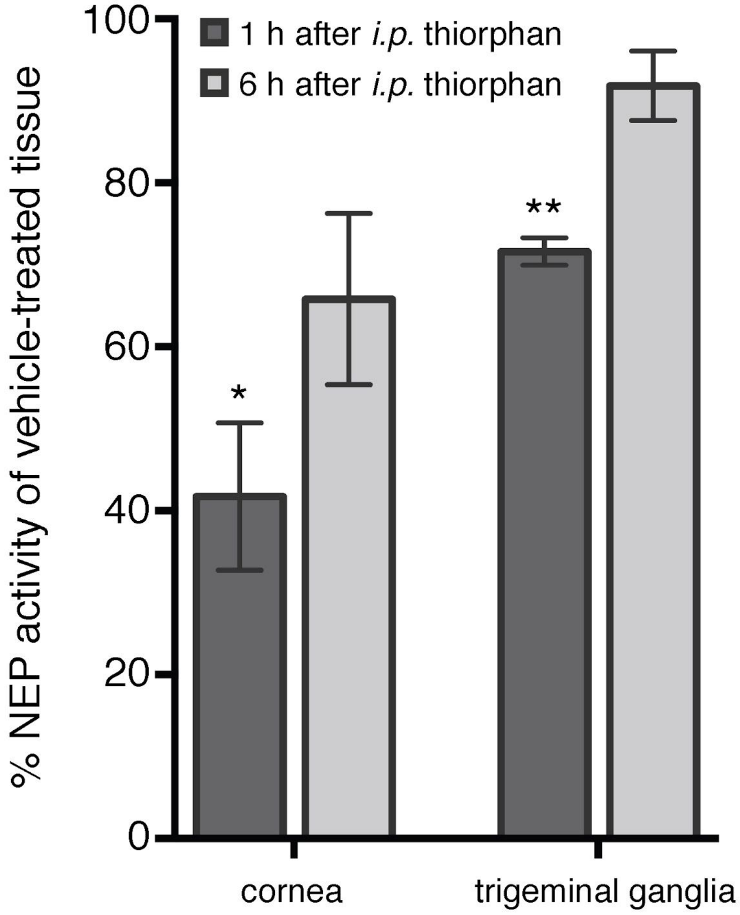

Supplementary Figure 3. Effect of thiorphan on NEP activity in the cornea and trigeminal ganglia. NEP activity in whole cornea and trigeminal ganglia isolated from uninjured WT mice at 1 and 6 h after a single intraperitoneal injection of 15 mg/kg thiorphan. Presented as % NEP activity of control, vehicle-treated tissue. In both tissues, NEP activity was significantly lower than in vehicle-treated tissue at 1 h after administration, but not at 6 h after administration of *i.p.* thiorphan (mean ± SEM; *P = 0.0226; **P = 0.002; one-way ANOVA with Bonferroni correction).

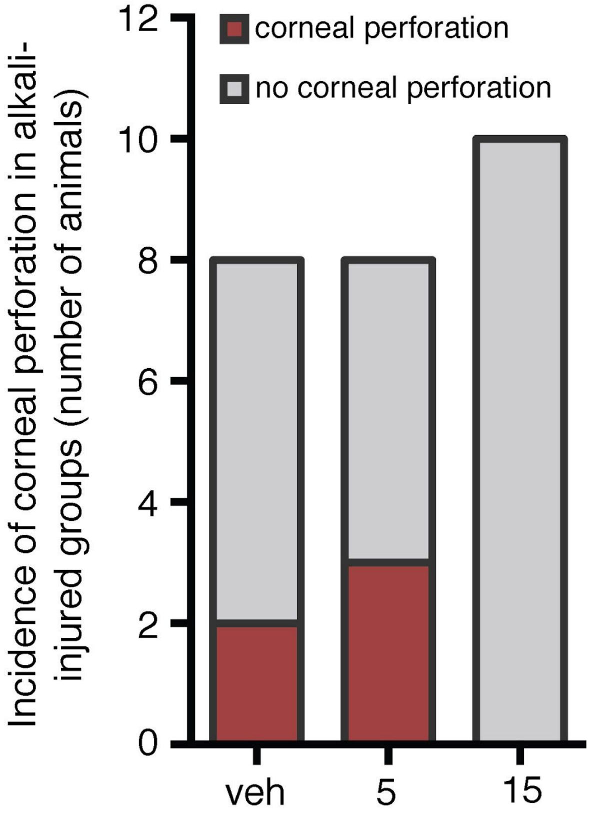

Supplementary Figure 4. Corneal perforation in alkali-injured WT mice. Incidence of corneal perforation by day 7 post-injury. Veh, vehicle; 5, 5 mg/kg thiorphan; 15, 15 mg/kg thiorphan.

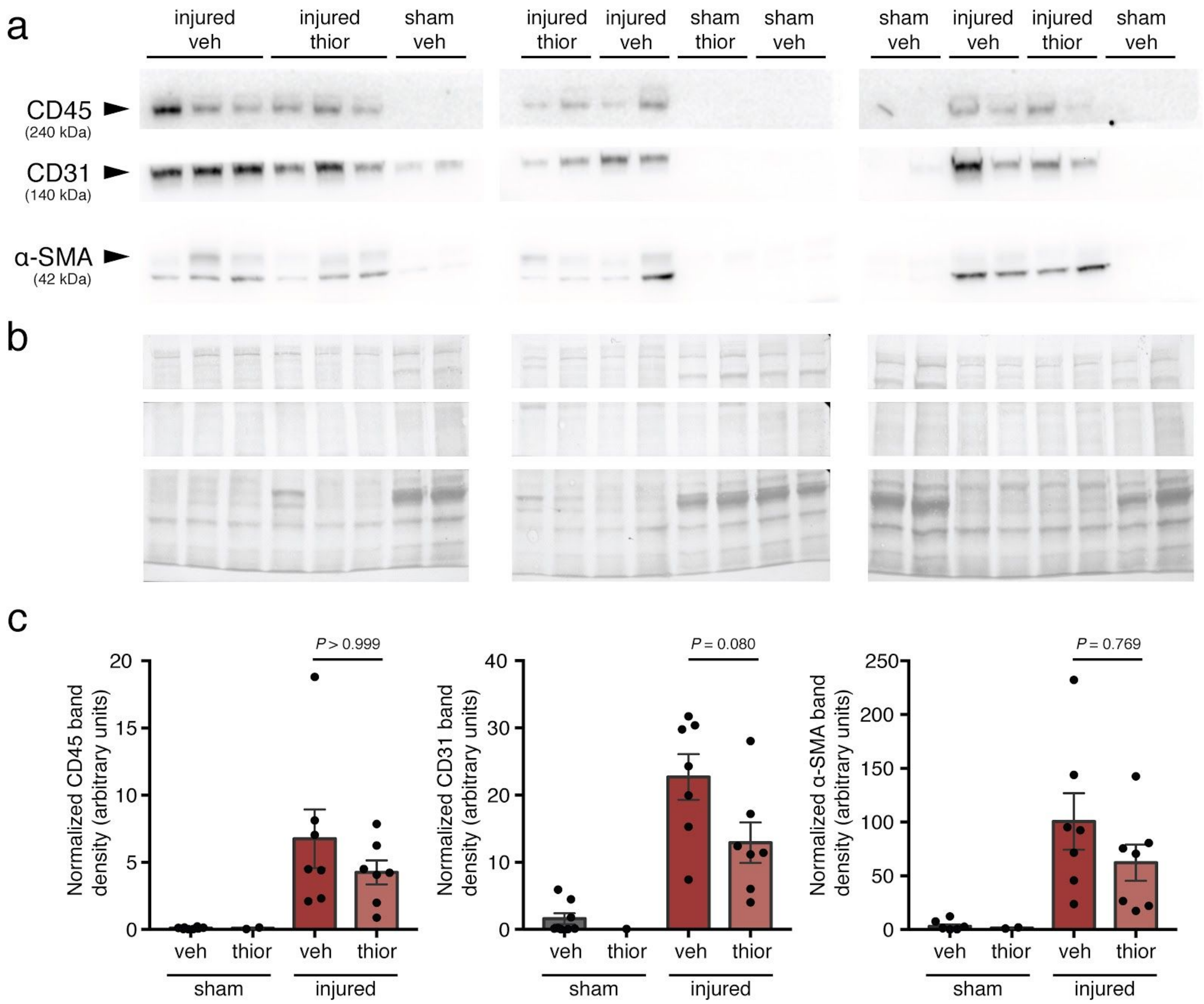

Supplementary Figure 5. Thiorphan administration does not significantly affect CD45, CD31, or α-smooth muscle actin expression in alkali-injured WT corneas at one week. (**a**) Immunoblots showing expression of CD45, CD31, and α-smooth muscle actin in whole cornea lysates from WT mice at one week post-alkali or sham injury. Samples from each experimental group were randomized among three gels that were processed simultaneously. (**b**) Amido black stain for total protein on membranes corresponding to those shown in (**a**), utilized as a loading control. (**c**) Band intensities normalized to total protein and intermembrane reference protein. Each point represents expression in lysate from a single cornea (mean ± SEM; one-way ANOVA with Bonferroni correction). SMA, α-smooth muscle actin; veh, vehicle; thior, thiorphan (15 mg/kg).

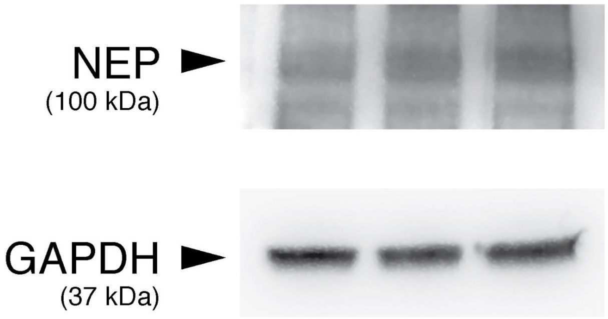

Supplementary Figure 6. Expression of NEP protein in TKE2 cell line. Immunoblot of NEP (100 kDa) in lysates from confluent TKE2 cultures, with glyceraldehyde 3-phosphate dehydrogenase (GAPDH, 37 kDa) run as a loading control. Membrane was cut at 50 kDa.

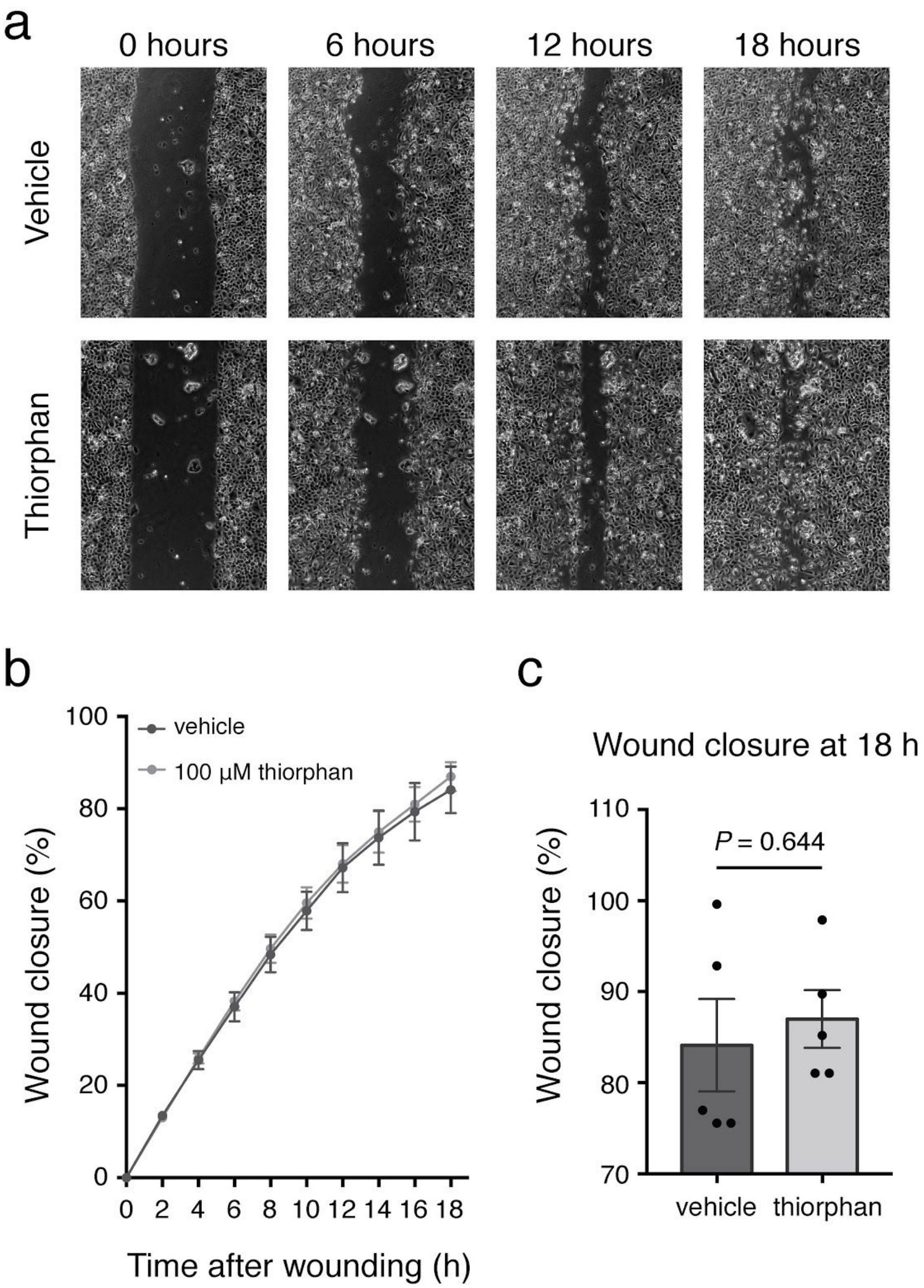

Supplementary Figure 7. Thiorphan does not affect migration of TKE2 cells after wounding *in vitro*. (**a**) Representative images from *in vitro* scratch assays demonstrating that cell migration into the cell-free wound region does not vary with thiorphan treatment. (**b**) Summary graph of the course of wound healing over the duration of the assay. (**c**) Quantification of wound closure at the final timepoint (n = 5 wells per condition; mean ± SEM; two-tailed *t* test).

Supplementary Methods

NEP enzyme activity following thiorphan administration. Uninjured WT mice were administered a single dose of *i.p.* 15 mg/kg thiorphan or vehicle (5% ethanol in saline) with a 28 gauge U-100 insulin syringe (Becton-Dickinson, Franklin Lake, NJ, USA). Mice were decapitated under deep isoflurane anesthesia 1 or 6 h after treatment for collection of bilateral trigeminal ganglia and corneas. Tissue was frozen in liquid nitrogen and stored at -80°C.

Frozen corneas were homogenized in 100 µL of 0.5% NP-40 lysis buffer in two 15 s bursts with 30 s on ice between bursts. Frozen trigeminal ganglia were homogenized in 200 µL of buffer for 10 s. Aprotinin (5 µg/mL; Thermo Scientific) and phenylmethylsulfonyl fluoride (200 µM; Thermo Scientific) were added to the buffer to prevent protein degradation. Samples were centrifuged at 10,000 g for 5 min at 4°C, and pellets were discarded. Total protein in each supernatant was determined by BCA assay according to manufacturer's instructions (Thermo Scientific).

Immunoblot analysis of WT corneas after alkali injury. Anesthetized WT mice were unilaterally injured with a 5 µL drop of 0.5 M NaOH on the left ocular surface for 30 s. Saline served as a sham injury. Subcutaneous meloxicam (2 mg/kg) was administered in sham and injured groups immediately after injury and 24 h after injury. Mice received *i.p.* 15 mg/kg thiorphan or vehicle (5% ethanol in saline) within 1 h after corneal injury and each day thereafter until euthanasia at the end of one week. In order to avoid any effects of manipulation on the ocular surface, mice used for immunoblot analysis were not imaged.

Freshly enucleated eyes were trimmed at the sclerocorneal limbus. The left cornea from each mouse was homogenized (VWR 200 Homogenizer) in 100 µL of cold RIPA buffer containing protease/phosphatase inhibitors. Three gels were required to represent all experimental groups. Corneal lysates containing equal amounts of protein (9.5 µg), determined by BCA assay, were randomized among the three 7.5% Mini-PROTEAN TGX precast polyacrylamide gels (BioRad), separated by electrophoresis, and transferred to nitrocellulose membranes with the BioRad Trans-Blot Turbo Transfer system. An equal concentration of intermembrane (IM) reference sample (pooled TKE2 lysate) was run on each gel in the same location. Membranes were cut at 50 and 150 kDa and blocked in 5% skim milk in TBST buffer at RT for 1 h. The upper third of each membrane (> 150 kDa) was immunoblotted in rabbit anti-CD45 (ab208022; Abcam) at 1:1000, the middle third (50 - 150 kDa) in rabbit anti-CD31 (ab28364; Abcam) at 1:250, and the bottom third (< 50 kDa) in mouse anti-alpha smooth muscle actin (SMA; ab7817; Abcam) at 1:500 overnight at 4°C. The IM reference lane was incubated in rabbit anti-neurokinin-1 receptor (NK1R; ab183713; Abcam) at 1:1000. After washing in TBST, all membranes were incubated with HRP-conjugated secondary antibodies (Abcam) at 1:5000

for 1 h at RT and developed with SuperSignal West Femto Maximum Sensitivity Substrate. Chemiluminescence was detected using a BioRad ChemiDoc XRS+ system with Image Lab software.

Following chemiluminescent detection, membranes were stained for total protein using amido black according to a published protocol[60]. In brief, membranes were washed in dH2O and submerged in 0.01% (w/v) amido black 10B (Abcam) in 10% acetic acid for 1 min. Membranes were then destained in 5% acetic acid, washed in dH2O, and dried at RT. Dried membranes were imaged using the Epi-White illumination setting with identical exposure times on the ChemiDoc XRS+ system.

For relative quantification, the integrated optical density value (defined as the sum of each background-subtracted pixel value) was determined for equal-sized boxes drawn around bands for each antibody in Image Studio Lite software (LI-COR Biosciences, Lincoln, NE, USA). Median background was calculated from perimeter values around each band of interest. As a loading control, the total amido black signal was determined for equal-sized strips centered in each lane, rather than a rectangle encompassing the entire stained area, to minimize errors due to lane bending[61]. The integrated optical density for each protein of interest was first normalized to the total amido black signal in its corresponding lane, and then to the normalized integrated optical density of the IM control protein, NK1R, from its corresponding membrane to control for intermembrane variability. NK1R bands were similarly normalized to the total amido black signal in corresponding lanes.

Samples were re-randomized and immunoblots were repeated with statistically similar results. Normalized data from the second set of immunoblots are presented.

Immunoblot analysis of TKE2 cells. TKE2 cells (ECACC 11033107; Sigma-Aldrich, St. Louis, MO, USA), a line of mouse corneal epithelial progenitors isolated from a CD-1 female mouse, were harvested at passage 3, incubated in 150 µL cold RIPA buffer with protease and phosphatase inhibitors (Halt Cocktail; Thermo Scientific), and sonicated on ice. After centrifugation at 13,000 g for 5 min at 4°C, the supernatant was collected and protein concentration was determined by BCA assay according to manufacturer's instructions.

Lysates containing equal amounts of protein (20 µg) were separated on a 10% Mini-PROTEAN TGX precast polyacrylamide gel (BioRad) and transferred to a nitrocellulose membrane with the BioRad Trans-Blot Turbo Transfer system. For detection of NEP and the loading control glyceraldehyde 3-phosphate dehydrogenase (GAPDH), membranes were cut at 70 kDa and blocked in 5% milk in TBST buffer at RT for 1.5 h. The upper half of the membrane (> 70 kDa) was incubated in goat anti-CD10 (NEP; PA5-47075; Invitrogen) at 1:1000, and the lower half (< 70 kDa) was incubated in mouse anti-GAPDH (MAB374; EMD Millipore) at 1:1000, both overnight at 4°C. After washing in TBST, membranes were incubated with HRP-conjugated secondary antibodies (Abcam) at 1:5000 for 1 h at RT and developed with SuperSignal West Femto Maximum Sensitivity Substrate (Thermo Scientific).

Chemiluminescence was detected using a BioSpectrum 810 imaging system with CCD camera (Ultra-Violet Products).

TKE2 *in vitro* scratch assays. Scratch assays with TKE2 cells between passages 3 and 10 were used to determine if thiorphan could promote wound closure *in vitro*. Cells were plated at a density of 62,500 cells/cm^2 and grown to confluence on uncoated 12-well Corning CellBIND plates (Corning Life Sciences, Corning, NY, USA) in Stemline Keratinocyte Medium II (S0196) with Stemline Keratinocyte Growth Supplement (S9945) and 2 mM glutamine (Sigma). The confluent monolayer was scratched with a 200 μL sterile pipette tip guided by a plastic ruler. Scratch-wounded monolayers were washed with prewarmed media to remove detached cells and debris before incubating in fresh media containing 1, 10, or 100 μM thiorphan (diluted from a 2 mM stock in water) or 10 μL of UltraPure water (Invitrogen, Carlsbad, CA, USA) in 0.5 mL of complete medium for 18 h. Conditions were assayed in triplicate wells, assigned at random on each plate.

Wound closure was monitored with time-lapse differential interference contrast (DIC) imaging on a custom-built Olympus IX-81 inverted microscope (Waltham, MA, USA) with a humidified, carbogenated (flow rate 0.5 - 1.0 L/min), 37°C environmental chamber at the University of Iowa Central Microscopy Research Facility. The motorized stage controller (ProScan II) and associated Olympus cellSens software allowed imaging of three pre-selected, “memorized” positions within each well. Images were at 4X every 15 min for the duration of the experiment. Scratch wound closure was analyzed in ImageJ software (NIH). The initial wound area (time 0) and remaining wound areas at 2 h intervals were manually outlined for quantification. Data are presented as average percent closure relative to initial wound area at each timepoint.
